# Unexpected Help: Follicular Regulatory T Cells in the Germinal Center

**DOI:** 10.3389/fimmu.2018.01536

**Published:** 2018-07-02

**Authors:** Markus M. Xie, Alexander L. Dent

**Affiliations:** Department of Microbiology and Immunology, Indiana University School of Medicine, Indianapolis, IN, United States

**Keywords:** T follicular regulatory cells, germinal center, follicular helper T cell, regulatory T cells, T cell differentiation

## Abstract

Follicular helper T (Tfh) cells are necessary for germinal center (GC) formation and within the GC, provide key signals to B cells for their differentiation into plasmablasts and plasma cells that secrete high-affinity and isotype-switched antibody (Ab). A specialized subset of Foxp3^+^ T cells termed T follicular regulatory (Tfr) cells, also regulate the differentiation of Ab-secreting cells from the GC. Tfr-cell function in the GC is not well understood, however, the dominant paradigm currently is that Tfr cells repress excessive Tfh and GC B cell proliferation and help promote stringent selection of high-affinity B cells. A mouse model where the Bcl6 gene is specifically deleted in Foxp3^+^ T cells (Bcl6FC mice) allows the study of Tfr cell function with more precision than other approaches. Studies with this model have shown that Tfr cells play a key role in maintaining GC B cell proliferation and Ab levels. Part of the mechanism for this positive “helper” effect of Tfr cells on the GC is Tfr cell-derived IL-10, which can promote B cell growth and entry into the dark zone of the GC. Recent studies on Tfr cells support a new paradigm for Tfr cell function in the GC reaction. Here, we review studies on Tfr cell functions and discuss the evidence that Tfr cells can have a major helper role in the GC-dependent Ab response.

## Introduction

A major function of the adaptive immune response is to produce highly specific antibodies (Abs) that bind to antigen (Ag) with high affinity and help to eliminate pathogens and foreign substances. A specialized subset of differentiated CD4 T cells, follicular helper T (Tfh) cells, are required in the germinal center (GC) reaction to help B cells generate high-affinity Abs to Ag (1, 2). Tfh cells control the initiation as well as the outcome of the GC B cell response (3–6). Tfh cells are critical for the proper production of protective Abs during an infection, however, the over-production of Tfh cells can also lead to autoimmunity since Tfh cells can help B cells to produce self-reactive Abs (6–8). Thus, the proper regulation of Tfh cell differentiation is essential both for normal immune function and for preventing autoimmune disease.

**Figure 1:**
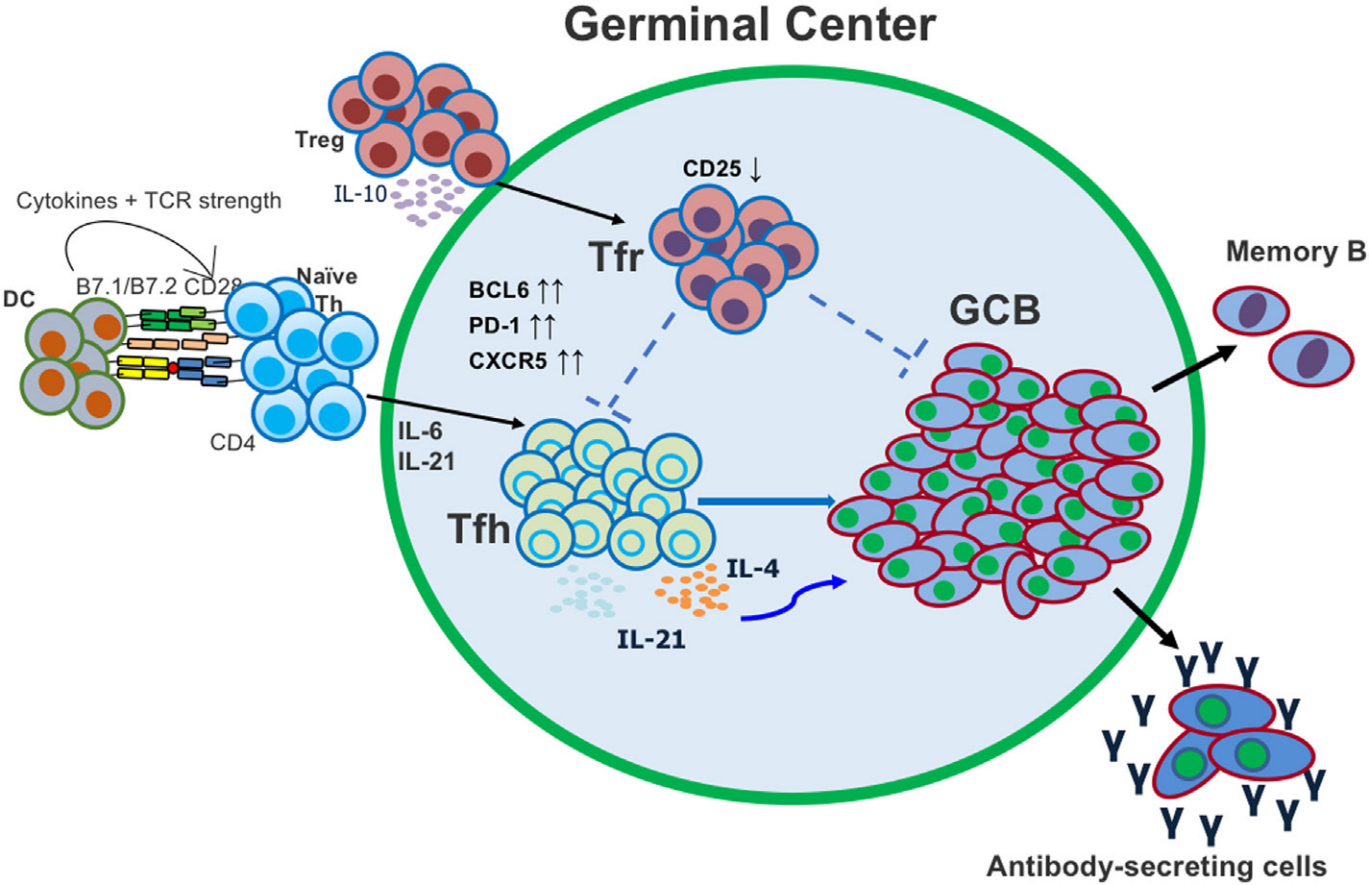
Follicular helper T (Tfh) and T follicular regulatory (Tfr) cells both act in the germinal center (GC) to regulate the generation of antigen (Ag)-specific antibody-secreting cells. Tfh cells differentiate from conventional CD4 T cells after activation with Ag and dendritic cell (DC) presentation. Tfr cells differentiate from conventional regulatory T cells (Tregs) and migrate into the GC.

Germinal center B cell responses are also regulated by T follicular regulatory (Tfr) cells, which develop from regulatory T cells (Tregs) and localize to the GC (9–16) (Figure 1). Tfr cells are generally thought to limit the function of Tfh cells in the GC (9–13, 16). Tfr cells, like Tfh cells, are dependent upon the transcriptional repressor protein Bcl6 for their development, but unlike Tfh cells express the canonical Treg master regulatory transcription factor Foxp3 (9–16). The prevailing model for Tfr cell function currently is that Tfr cells repress excessive Tfh and GC B cell proliferation and promote the selection of high-affinity B cells (9–13, 16), however, the complete range of Tfr cell functions are poorly understood. A Tfr-deficient mouse model where the *Bcl6* gene is specifically deleted in Foxp3^+^ T cells (*Bcl6* fl/fl *F*oxp3-*C*re or Bcl6FC mice) has been used by us and others to study Tfr cell function in immune and autoimmune responses (14, 17–19). With this model, we and others have unexpectedly found that the major Tfr cell function may not be inhibiting the GC response but instead helping promote the Ab response and even the magnitude of the GC response. Here, we discuss the current understanding of the differentiation and physiological functions of Tfr cells. We also discuss how Tfr cells balance suppressive and helper functions, the potential mechanisms underlying Tfr cell functions, and directions for future investigations.

## Differentiation and Regulation of Tfr Cells

**Figure 2:**
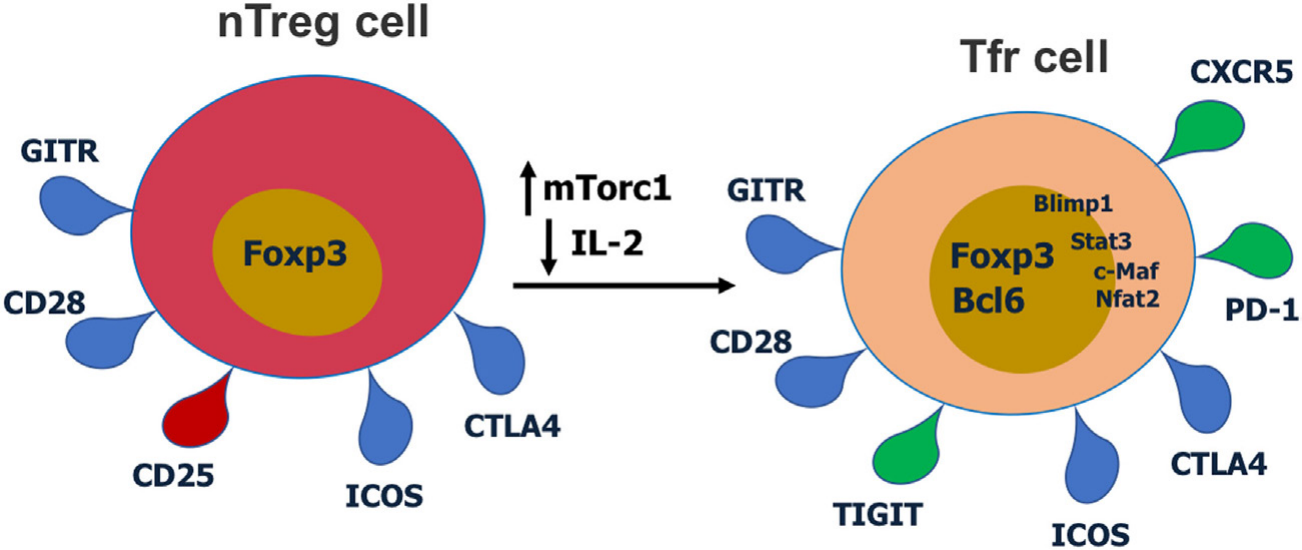
Cell surface receptors and transcription factors involved in T follicular regulatory (Tfr) cell differentiation and function. Red receptors are downregulated and green receptors are upregulated during Tfr cell differentiation.

Several studies have shown that Tfr cells primarily differentiate from Foxp3^+^ Treg precursor cells (10, 11, 20–22) (Figure 2), however, like Tfh cells, Tfr cells can also develop from naïve CD4 T cells (23). Tregs are generated either during T cell differentiation in the thymus (tTregs) or from mature CD4 T cells in the periphery (pTregs) (24, 25), but whether Tfr cells preferentially develop from tTregs or pTregs is not known. Tregs in the intestinal mucosa are predominantly pTregs that develop to Ags derived from microbiota and diet as a tolerance mechanism (24, 25). Tfr cells that develop in the gut lymphoid tissues such as Peyer’s patches may therefore differentiate from pTregs, and so ultimately may have a naïve CD4 T cell origin. Interestingly, Peyer’s patch Tfr cells have a markedly different transcriptome than peripheral lymph node Tfr cells, possibly suggesting a different origin (26).

T follicular regulatory cells express Tfh cell surface markers such as PD-1, CXCR5, and ICOS, Treg surface markers such as CTLA-4 and GITR, and the master regulatory transcription factors for both Tregs (Foxp3) and Tfh cells (Bcl6) (9–11, 14, 27). Thus, Tfr cells display a hybrid or mixed Tfh/Treg phenotype. Most studies have analyzed Tfr cells in the mouse, but phenotypically similar Tfr cells have also been described for humans (28, 29) and macaques (30). Tfr cells also express significant levels of Blimp1, a transcriptional repressor protein that suppresses Bcl6 expression (10, 31). Notably, Tfr cells express lower CD25 compared with non-Tfr Tregs (14, 29). Together with Bcl6, Nfat2 upregulates CXCR5 expression on Tregs and enables them to migrate to GC, take on the follicular phenotype and become Tfr cells (9–11, 14, 32). Recent work has revealed that the mTor pathway is a key regulator of Tfr cells. The mTorc1 complex is essential in regulating the conversion of Tregs to Tfr cells and this is potentially through a Stat3–Tcf-1–Bcl6 pathway (33, 34). Our lab has also specifically found that in contrast to Tfh cells which can develop in the absence of Stat3, Stat3 is essential for Tfr cell development (15). Deletion of *Pten* in Tregs leads to upregulated mTorc2 activity and heightened Tfr cell development (35). Thus, the Akt–mTor2 kinase pathway promotes Tfr cell development and the Pten phosphatase helps restrain excessive Tfr cell development (35).

Antigen exposure triggers the differentiation of Tfr cells and this process is dendritic cell (DC)-dependent (10, 11, 23, 27). Sage et al. used mice that express diphtheria toxin receptor specifically on DCs to test this (12). DC-depletion led to substantially decreased Tfr cells, however, it is unknown which specific DC subsets directly contribute to Tfr cell differentiation. At the same time, PD-1-ligand expressed on DCs has an inhibitory role on Tfr cell development (36). Tregs can repress the function of Ag presenting cells (APCs) including DCs (37), but whether Tfr cells can affect DCs or other APCs and how this might affect the GC response is unknown. Precisely what Ags and signals that Tregs respond to in order to become Tfr cells is not well understood. Tfr cells respond more strongly to self-Ags than foreign Ags, which fits with the self-reactive nature of tTregs (23, 38). While Tfr cells can be found that have specificity for the immunizing Ag (23), a recent study on the TCR specificity of Tfh and Tfr cells indicated that in contrast to Tfh cells, Tfr cells do not respond well to the cognate Ag after immunization (22). Furthermore, an analysis of TCR gene sequences in Tfh and Tfr cells indicated that Tfh cells are a sub-population of cells related to naïve CD4 T cells, whereas Tfr cells showed a TCR profile very similar to the total Treg population (22). These findings are consistent with the model that Tfh cells are Ag-specific T cells that proliferated after Ag stimulation, while Tfr cells develop in a polyclonal and Ag-independent manner from Tregs. Therefore, Tfr cells either develop from Tregs in a polyclonal TCR-dependent response involving recognition of self-Ag, or Tfr cells expand and differentiate by an Ag-independent and TCR independent pathway [e.g., Jagged1 plus Ox40 stimulation (39)]. Note that the Maceiras et al. study (22) of Tfr cell TCR sequences analyzed Tfr cells from peripheral LNs, and the TCR specificity of Peyer’s patch Tfr cells may be more similar to naïve CD4 T cells that are responsive to gut Ags.

T cell co-stimulation is required for Tfr cell differentiation as either CD28 or ICOS deficiency leads to reduction of Tfr cells (10, 27, 40). Mice with CD28 deficiency specifically in Tregs (using Foxp3-cre) had a large reduction in Tfr cells in the draining lymph node after NP-OVA immunization (40). This is largely due to the roles of CD28 in inducing Foxp3 expression as well as Tfr cell proliferation (10, 41–44). Similarly, Tfr cell development is abrogated in ICOS-deficient mice (27). ICOS signaling modulates the expression of Bcl6 and c-Maf in Tfh cells and might play a similar role in Tfr cells (45–47). Bcl6 is an essential transcription factor for Tfr cells, and recent studies suggest that c-Maf is also pivotal for Tfr cell differentiation (10, 11, 14, 48, 49). Bcl6 and Blimp1 reciprocally repress expression of the other factor in both Tfh and Tfr cells (31, 50). The regulation of Tfh cell differentiation by Blimp1 is Bcl6-dependent while Blimp1 controls Tfr cell differentiation independent of Bcl6 (31). One mechanism for Bcl6-independent Blimp1 activity may relate to regulation of Nfat2, which has been shown to be important for upregulation of CXCR5 on Tfr cells as well as for expression of PD-1 (32, 51). Blimp1 has been shown to repress Nfat2 expression (51), and thus Blimp1 could have a suppressive role for CXCR5 and PD-1, both of which are key genes increased in Tfr cells. Increased expression of Nfat2 in Blimp1-deficient Tregs could then lead to Bcl6-independent expression of CXCR5 and PD-1, and appearance of Tfr-like cells (31). Tfr cells were repressed by high IL-2 levels at the peak of influenza infection and this was through a Blimp1-dependent mechanism (19). IL-2 is also a negative signal for Bcl6 expression, and decreased IL-2 promotes induction of Tfr cells. After the peak anti-flu virus immune response, CD25 expression is downregulated in some Tregs while Bcl6 is increased, leading to Tfr programming (19). Thus, IL-2 is a key factor regulating Tfr differentiation, promoting Blimp1 expression while repressing Bcl6 in Tregs to preclude Tfr cell development.

PD-1, which is expressed by both Tfh and Tfr cells, inhibits Tfr differentiation and their suppressive function (10, 11, 16, 27). Sage et al. showed that Tfr cells in *Pdcd1-*deficient mice had greater suppressive function and resulted in decreased Ab production both *in vitro* and *in vivo* (27). The exact mechanism for the increased inhibitory function of *Pdcd1-*deficient Tfr cells remains unclear. At the same time, PD-1 ligand is required for Tfr cell generation, however, it is not clear if this is a direct or indirect effect on Tfr cells (23). Similarly, CTLA-4, the inhibitory receptor which binds to CD80 and CD86, limits the differentiation of Tfr cells (13, 52, 53). However, restricted CTLA-4 deficiency in Tregs contributes not only to enhanced Tfr cells but also enhanced Tfh, GCB cells, and Ab responses (53). One explanation is that in the absence of CTLA-4 function in Tregs, there is uncontrolled inflammation that drives higher Tfh cell and GCB responses. However, since it is not clear what drives the enhanced Tfh, GCB, and IgE responses, a “helper” role of Tfr cells cannot be completely excluded (53). Deletion of CTLA-4 results in increased IL-10 production by Tregs (54). Since IL-10 can promote GC responses (17, 55), it is possible that increased IL-10 production by Tfr cells contributes to the increased GC and Ab response in CTLA-4 KO mice.

The majority of research on Tfr cells has been conducted in the mouse system but a few recent studies have elucidated Tfr cell populations in human GCs that are basically similar to Tfr cells in mice (29, 56, 57). CXCR5^+^ Tfh-like cells in blood, also known as circulating Tfh (cTfh) cells, are typically used as a proxy marker for the GC Tfh cell response in humans. By assessing cTfh cell frequency in patients with monogenic mutations leading to immunodeficiency, a large number of genes controlling human Tfh cell development have been categorized (58). Circulating Tfr (cTfr) cells in blood are also used as a correlate of the Tfr cell response (28, 59–62), however, in contrast to Tfh cells (58), relatively few genes that control Tfr cell development and function in humans have been characterized to date (e.g., *LRBA* and *CTLA4*) (62–64). Thus, much work remains in fully understanding specific genes and pathways that regulate human Tfr cells.

## Suppressive Functions of Tfr Cells

T follicular regulatory cells have been described in the literature mainly as suppressors of the GC reaction and the Ab response, repressing the proliferation of Tfh cells and GC B cells, and limiting the generation of Ab-secreting cells and overall Ab responses. However, the experimental approaches taken in many studies can give rise to alternative interpretations. *In vitro*, Tfr cells can suppress the proliferation and cytokine production of Tfh cells as well as the proliferation and Ig secretion of B cells, similar to the *in vitro* suppressive function seen with non-Tfr Tregs (13, 27–29, 38, 65). *In vivo* studies have demonstrated that Tfr cells, analyzed initially by depletion of total Tregs, can suppress the numbers of GC B cells and Tfh cells (9–11, 13, 27, 53). However, these studies may not represent specific effects of Tfr cell depletion or physiological Tfr cell function. Total Treg deletion (10, 11, 13, 66) provokes severe inflammation and causes a very broad effect on T cell responses, thus obscuring the specific functions of Tfr cells. Studies using adoptive transfer of Tfr cells along with other T cells into T cell-deficient mice or Tfh-cell-deficient mice might have non-physiological effects due to the abnormal immune environment of the recipient mice (10, 11, 27, 33). Studies where Tfr cell numbers are greatly enhanced due to deletion of Roquin (34), or where Tregs are forced to migrate to the B cell follicle by ectopic CXCR5 expression (32) might also lead to non-physiological suppression and/or non-specific suppression of GC responses. Mice with the Nfat2 gene deleted in Tregs with Foxp3-cre showed a partial loss of Tfr cells and augmented numbers of GC B cells, Tfh cells, and Ag-specific Abs after immunization (32). However, a more general loss of Treg function by loss of Nfat2 affecting Tfh cell expansion cannot be discriminated from the specific effects from loss of Tfr cells. *In vitro* studies of Tfr cells cannot mimic the complex *in vivo* environment of the GC reaction and cannot analyze affinity selection of GC B cells. Together, a re-interpretation of the Tfr cell literature helps to explain why the function of Tfr cells assessed using Bcl6FC mice (14, 17, 19), is strikingly different from many other studies on Tfr cell function.

Nonetheless, it is clear that under some conditions, Tfr cells can negatively regulate the GC reaction, and the precise mechanisms that Tfr cells use to negatively regulate the GC is one of the unsolved mysteries in the Tfr cell field. Tregs can suppress immune responses by multiple known mechanisms: IL-2 consumption, secretion of inhibitory factors (IL-10, TGF-β, IL-35, granzyme B, CD39, CD73, and TRAIL), and CTLA-4-mediated inhibition of Tfh cell co-stimulation (67–69). Of these known suppressor factors, we can narrow down mechanisms for Tfr cells based on previous data. Tfh cell differentiation is inhibited by IL-2 (70–72), and IL-2 consumption by Tfr cells could be predicted to help stabilize Tfh cell responses. However, Tfr cells have low levels of the high-affinity IL-2 receptor CD25 (14), which indicates a lessened capacity to compete for available IL-2. IL-10 is unlikely to be the key suppressor factor, since IL-10 is a stimulatory or growth factor for GC B cells (17), and furthermore, IL-10 expression by Tfh cells is increased in the absence of Tfr cells in Bcl6 mice (14). IL-35 is unlikely to be a Tfr suppressor factor as it primarily affects T cell proliferation (73), and data with Bcl6FC mice does not indicate an effect of Tfr cells on the number of Tfh cells (14). Granzyme B is unlikely as a major mechanism as it is decreased in Tfr cells compared to Tregs (10). Metabolic suppressor pathways such as CD39 and CD73 have not been extensively characterized and are possible effectors of suppression by Tfr cells as they could potentially affect cell proliferation in the GC. In mice, TGF-β is known to stabilize Tfh cell responses (74), and prevent excess Tfh cell responses (75). In humans, TGF-β is required for Tfh cell differentiation (76). A lack of TGF-β signaling from loss of Tfr cells does not clearly explain the normal Tfh cell numbers in the presence of increased Tfh cytokine expression in Bcl6FC mice (14). TRAIL is cytotoxic to follicular B cell lymphomas, which have a GC phenotype (77), but otherwise there is no data about TRAIL activity in GCs, particularly regarding Tfr cells. CTLA-4 expression by Tfr cells may inhibit the ability of Tfh cells to receive key co-stimulation signals from GC B cells, thus limiting Tfh cell and thus Tfh cell-driven GC B cell expansion. Unfortunately, studies on the role of CTLA-4 function in Tfr cells are difficult to interpret, as noted above (13, 52, 53).

A recently described mechanism for Tfr cells to inhibit Tfh cells and the GC is secretion of a decoy IL-1 receptor that inhibits Tfh cell differentiation (38). This pathway appears to be most critical during early Tfh cell activation and differentiation rather than during the GC reaction itself. Furthermore, data pointing to this decoy IL-1 receptor pathway being used specifically by Tfr cells to control Tfh cells *in vivo* is lacking. Another potential pathway used by Tfr cells to control Tfh cells and GC B cells is the inhibitory receptor TIGIT, that is important for Treg suppressive function (14, 78). Intriguingly, the two major suppressive pathways utilized by TIGIT^high^ Tregs are IL-10 and Fibrinogen-like protein 2 (Fgl2) (78). Fgl2 is a secreted protein that binds the inhibitory IgG receptor FcγRIIB (79). As noted above, it is unlikely that loss of IL-10 from Tfr cells contributes to the deregulated cytokine expression of Tfh cells. Thus, Fgl2 may be a key factor used by Tfr cells to regulate GC B cells. Interestingly, FcγRIIB KO mice are known to develop lupus (80), and Fgl2 KO mice develop glomerulonephritis, a pathologic manifestation of auto-Abs in severe lupus disease also seen in IgA nephropathy (81, 82). Fgl2 KO mice have Treg defects, but the GC response in these mice has not been characterized (81). TIGIT^high^ Tregs can also affect cell activation by inducing tolerogenic DCs *via* CD155 (83). But currently, data showing Tfr cells controlling the GC *via* TIGIT is lacking.

In analyzing mice with deletions of Bcl6 or Stat3 specifically in the Treg lineage (Bcl6FC and Stat3FC mice, respectively), we found noteworthy differences between how Tfr cells are regulated by Stat3 and Bcl6 (14, 15). While Tfr cells are strongly depleted in both Bcl6FC and Stat3FC mice (14, 15), there are significant differences in the phenotype. First, in Bcl6FC but not Stat3FC mice, Tfh cells produce higher levels of cytokines compared to control mice. Second, Ag-specific IgA is increased in Bcl6FC mice whereas Ag-specific IgG is increased in Stat3FC mice (14, 15). At the same time, Tfh cell and GC B cell numbers are not altered in either Bcl6FC or Stat3FC mice compared to control mice (14, 15). The function of Stat3 in Tfr cells is not understood. Analogous to Tfh cells (84, 85), Stat3 may be important for Tfr cell development by inducing Bcl6 expression in Tregs in response to cytokines such as IL-6 and IL-21. Stat3 expression is also activated in Tfr cells by the mTorc1 pathway (33). Bcl6 is required for the development of the CXCR5^+^PD-1^+^ follicular T cell phenotype, and the induction of Bcl6 by STAT factors may be essential for both Tfr cell as well as Tfh cell development. If this is the case though, why does deletion of Stat3 in Foxp3-expressing cells produce a different phenotype than deletion of Bcl6 in Foxp3-expressing cells? Why are cytokines upregulated from Tfh cells in Bcl6FC mice but not in Stat3FC mice? The answer to this question is currently unknown but is essential for fully understanding Tfr cell development and function. One possible explanation for the difference is that there is a larger population of residual Tfr cells in Stat3FC mice compared to Bcl6FC mice and these residual Tfr cells in Stat3FC mice are enough to negatively regulate Tfh cells. Thus, there is a greater deletion of Tfr cells in Bcl6FC mice, leading to a more complete loss of repression by Tfr cells, and thus increased Tfh cell activity. The increased Tfh cell cytokines in Bcl6FC mice might promote the elevated IgA response that is not seen in Stat3FC mice. In summary, in the Bcl6FC model, Tfr cells repress Tfh cell activity but not proliferation. Why Ag-specific IgG is increased in Stat3FC mice is unclear, but possibly the residual Tfr cells in Stat3FC mice have augmented GC helper activity.

T follicular regulatory cells have been studied extensively in immune responses induced by model protein Ags and adjuvants. Studies on Tfr cell function in regulating the gut microbiota (86) or in viral infection (17–19) have also been performed. More than the type of immune challenge, the model system used to assess Tfr cell function (e.g., Treg depletion in mice versus Bcl6FC mice) determines whether suppression by Tfr cells is observed.

## Role of Tfr Cells in Autoimmune Disease

An important area where Tfr cells have a clear suppressive effect on the GC and Ab response, even in Bcl6FC mice, is in suppression of auto-Abs that drive autoimmune disease (14, 18, 19, 32, 40). This role of Tfr cells in suppressing auto-Ab production was elucidated most thoroughly by Fu et al. who showed that Bcl6FC mice developed late-onset Sjogren’s-like autoimmune disease and autoimmunity could be induced in young mice by immunizing mice with salivary gland extracts (18). The precise mechanisms for how Tfr cells can suppress auto-Abs while at the same time promote the Ab response to foreign Ags remains unexplored. One possible explanation is that since Tfr cells, like Tregs, have a bias toward self-Ag recognition (22, 23, 38), they are able to inhibit self-reactive Tfh cells that might develop in the GC by competing with them for recognition of self-Ags on GC B cells and binding and blocking B7 co-stimulatory receptors *via* CTLA-4. Little is known about the role of Tfr cells in human autoimmune disease, but increased levels of cTfr cells are observed in patients with Sjogren’s disease (28, 59) and systemic lupus erythematosus (60). Interestingly, an increased ratio of cTfr to cTfh cells is strongly associated with more severe disease in the case of Sjogren’s syndrome (59). Whether high levels of cTfr cells simply represent the presence of active GC responses or whether cTfr cells are especially elevated in autoimmune disease is not clear. The data with Sjogren’s cTfr cells is particularly hard to interpret since the cells have an immature CD25^+^ Tfr phenotype and their relationship to GC-localized Tfr cells is unclear (59).

## “Helper” Functions of Tfr Cells

Although Tregs themselves are overwhelmingly described as suppressor cells, there are several reports that Tregs can promote immune responses in certain circumstances. Under inflammatory conditions or in mice with mutations in genes that affect Foxp3 expression, a fraction of Tregs can become “ex-Tregs” and differentiate into proinflammatory cells (87, 88). Surprisingly, ex-Tregs can also convert into functional Tfh cells in Peyer’s patches (89) and in atherosclerosis (90).

The first published characterization of Tfr cells in 2009 by Linterman et al. showed that Tfr cells had a key “helper” role in terms of helping Tfh cells select high-affinity Ag-specific B cell clones (7). In their proposed model, Tfr cells restrict the outgrowth of non-Ag-specific B cell clones in the GC, presumably allowing for more efficient interaction of Tfh cells with selection of specific high-affinity Abs (7). At the same time, the Linterman et al. data can also be interpreted as showing evidence for a major helper function for Tfr cells in the GC. For instance, a significant decrease in Ag-specific GC B cells is observed after total Treg depletion at the same time that total GC B cells increased (7). This can be interpreted as two distinct processes: (1) loss of Tfr cells leads to a loss of Tfr cell helper activity and thus reduced Ag-specific GC B cells and (2) because total Tregs are depleted, there is a massive increase in GC responses to commensals and self-Ags—responses that are normally inhibited by Tregs. Even though these latter commensal-specific and self-Ag-specific GCs may be weakened by loss of Tfr cell helper activity, the large number of these responses leads to a total increase in GC B cells.

A different Treg–Tfh helper pathway was shown by Leon et al., who found that Tregs are required for the normal anti-influenza Tfh cell response (66). In this study, ex-Tregs were not converting into Tfh cells, and Leon et al. proposed a mechanism where CD25^+^ Tregs take up IL-2 and limit the overall availability of IL-2, thereby promoting Tfh cell differentiation (66). Importantly, however, Leon et al. did not investigate loss of Tfr cells (which would occur with Treg depletion) as a mechanism for the Treg helper effect, and their data does not eliminate a helper role for Tfr cells in the Tfh/GC response in the virus infection system.

Because of the problems associated with deleting total Tregs and the lack of specific and robust models to deplete Tfr cells *in vivo*, we developed Bcl6FC mice (14). In these mice, Tfr cell development is specifically blocked without a loss in total Tregs or Treg function (14). We determined that loss of Tfr cells led to a significantly decreased IgG response and that Tfr cells were required to produce the highest affinity Ag-specific Abs (14). These results are consistent with a critical helper role for Tfr cells in the GC. In our published results, we did not observe a loss of GC B cells or Tfh cells in Bcl6FC mice despite the decreased IgG response (14). This could be due to the time-point where we analyzed the GC or the type of Ag used to induce the GC.

In 2017, Laidlaw et al. presented the clearest evidence to date that Tfr cells can act as essential helper cells in the GC (17). In this study, mice were infected with lymphocytic choriomeninigitis virus (LCMV) and the GC and Ab response analyzed (17). Importantly, Laidlaw et al. used Bcl6FC mice and Treg-specific IL-10 cKO mice to demonstrate that Tfr cells are a critical source of IL-10 in the GC and that IL-10 drives the growth of GCs by promoting entry of GC B cells into the dark zone (17). In the absence of IL-10-producing Tfr cells, GC B cell numbers and the LCMV-specific Ab response were decreased (17). A recent study with malaria infection in mice also showed that IL-10 was critical for the maintenance of the GC and GC-derived Ab response (55). Overall, these recent findings strongly support the idea that IL-10-producing Tfr cells have a major role in maintaining the GC reaction and thus act as “helper cells.” In our lab, we have been using Bcl6FC mice and analyzing the role of Tfr cells in a food allergy model with peanut Ag. In this model, we find that Tfr cells help maintain the peanut-specific GC response and IgE response (Markus M. Xie and Alexander L. Dent, manuscript in preparation). Tfr cells thus appear to have a key role in allergic immune responses, and represent a novel target for allergy-specific immunotherapy.

## Outstanding Questions and Future Directions

As of this writing, Tfr cells have only been analyzed in a very small fraction of infectious disease models and immunological diseases such as allergy and autoimmunity. Testing Tfr cell function in various disease states will be an important area for future research on Tfr cells. Also unknown is if Tfr cells affect diseases that are not driven by Ab-mediated pathology. Whether Tfr cells play a regulatory role in cancer, diabetes, heart disease, atherosclerosis, or other types of inflammatory diseases, is ripe for exploration. The mechanism of Tfr cell help in the GC is not completely understood and an important topic is why some types of GC responses seem to rely on Tfr cells for help whereas other GC responses are only mildly affected or not affected at all by loss of Tfr cells. A major issue for future studies is whether Tfr cells switch between help and suppression in the GC for foreign Ag, or primarily act as helpers for foreign Ag and suppressors of autoimmune responses. If Tfr cells act as suppressor cells of non-autoimmune responses in the GC, what mechanism of suppression do they use, and what controls whether Tfr cells act as suppressors versus helpers? Do human and mouse Tfr cells have similar helper and repressor functions? Tfr-like cells have been found circulating in both mouse and human; what is the relationship of these cells to Tfr cells in the GC? Also unclear is how Tfr cells regulate Ab affinity maturation and Tfh responses. A major question is whether Tfr cells regulate the generation or differentiation or survival of memory B cells. Finally, almost nothing is known about what signals drive Tfr cell responses to the GC and what Ags do they recognize? Thus, there are huge numbers of vital questions about Tfr cells that need to be answered through more research.

## Conclusion

**Figure 3:**
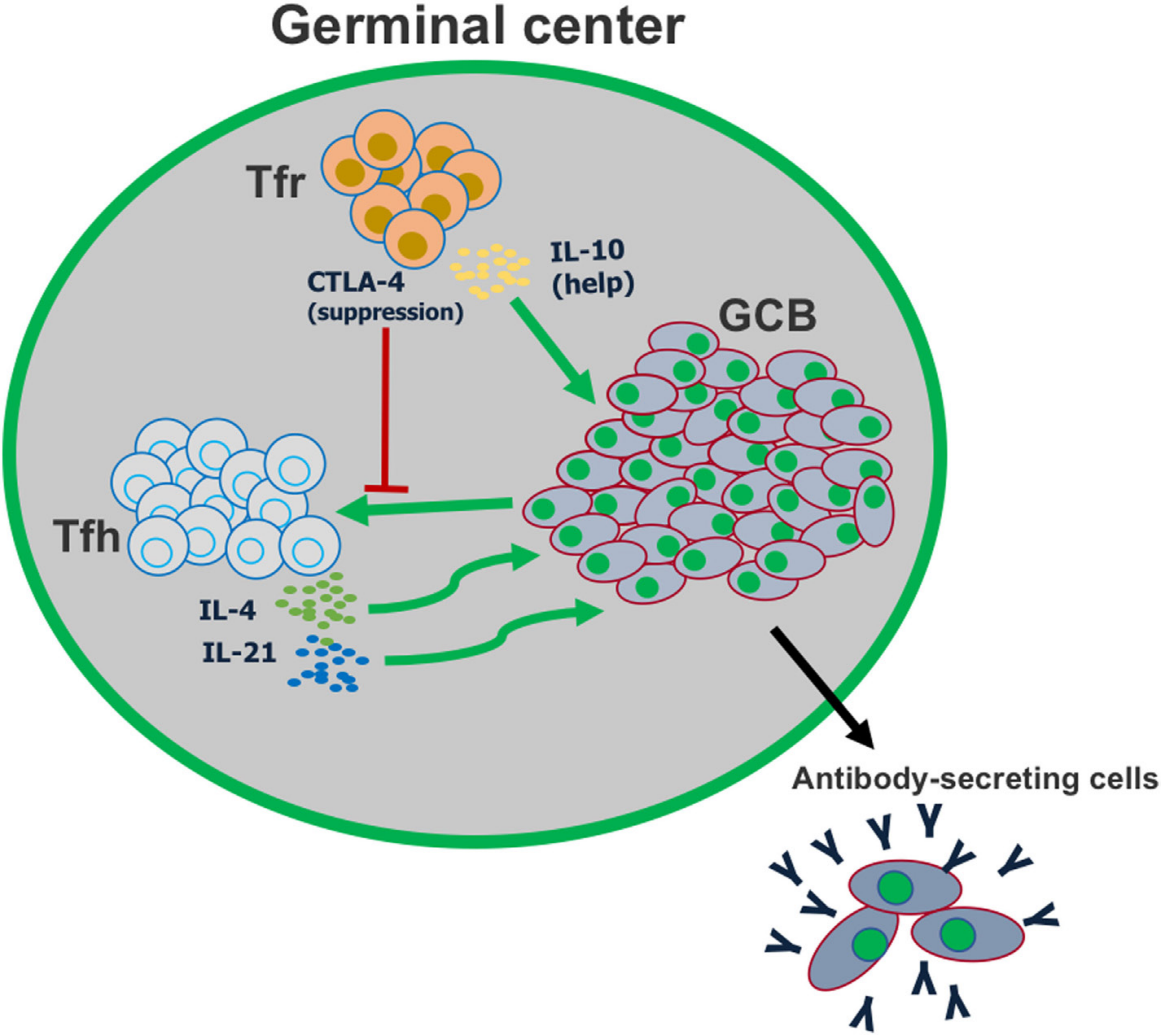
T follicular regulatory (Tfr) cells play a dual role in the germinal center reaction. Tfr cells can suppress the activation of follicular helper T (Tfh) cells, in part by CTLA-4-mediated inhibition of co-stimulation. However, Tfr cells also play a major role in helping promote germinal center B cell proliferation, by producing IL-10.

Even though Tregs act overwhelmingly as major suppressors of the immune response, Tfr cells provide a striking and clear example of Tregs acting as “helper” cells for the immune response. At least part of this Tfr cell helper function is producing IL-10 that promotes GC B cell growth and the GC-dependent high-affinity Ab response. Thus, in the context of the GC response, Tfr cells appear to maintain a key balance between help (GC maintenance, Ab response, and Ab affinity) and suppression (Tfh cell numbers, GC B cell numbers, Tfh cell cytokines, and auto-Abs) (Figure 3). One interesting idea is that the autoreactivity and suppressive capability of Tregs is used to help control autoimmunity in the GC but has been co-opted to also promote the overall GC response. Much work remains to fully understand the role of Tfr cells in the overall humoral immune response, and in the larger scope of the immune system.

## Author Contributions

AD and MX both wrote and made equal contributions to the manuscript.

## Conflict of Interest Statement

The authors declare that the research was conducted in the absence of any commercial or financial relationships that could be construed as a potential conflict of interest.
